# Probing spatiotemporally organized GPCR signaling using genetically encoded molecular tools

**DOI:** 10.1038/s12276-025-01485-2

**Published:** 2025-07-01

**Authors:** Yonghoon Kwon, Sohum Mehta, Jin Zhang

**Affiliations:** 1 Department of Pharmacology, University of California San Diego, La Jolla, CA USA; 2 Department of Life Sciences, Gwangju Institute of Science and Technology, Gwanju, Republic of Korea; 3 Integrated institute of Biomedical Research, Gwangju Institute of Science and Technology, Gwanju, Republic of Korea; 4 Shu Chien-Gene Lay Department of Bioengineering, University of California San Diego, La Jolla, CA USA; 5 Department of Chemistry and Biochemistry, University of California San Diego, La Jolla, CA USA; 6 Moores Cancer Center, University of California San Diego, La Jolla, CA USA

**Keywords:** Hormone receptors, Fluorescent proteins, Fluorescence imaging

## Abstract

G-protein-coupled receptors (GPCRs) control various downstream signaling pathways, with multiple effectors whose interactions are subject to sophisticated regulation to achieve signaling specificity. Spatiotemporal organization of GPCR signaling is essential for efficient control of multifaceted signaling pathways. To study how this spatiotemporal signaling is structured and affects cellular functionality, various genetically encoded molecular tools that can detect and perturb the target biochemical activities at a subcellular level have been developed. In this Review, we introduce various types of fluorescent protein-based biosensors and molecular tools that allow us to directly elucidate the spatiotemporal mechanisms of GPCR signaling regulation at a subcellular level. Finally, we highlight several applications of these molecular tools to study the spatiotemporal organization of GPCR in living cells to obtain a comprehensive understanding of the signaling architecture.

## Fluorescent tools illuminate subcellular GPCR signaling dynamics

G-protein-coupled receptors (GPCRs) are crucial for many body functions, such as sensing light and taste. Researchers focus on understanding GPCR signaling in different cell parts using special tools called biosensors. Biosensors are molecular tools that detect specific biological activities and convert them into visible readouts. The researchers used fluorescent biosensors to track GPCR signaling in real time within living cells. These biosensors help to visualize where and how GPCRs signal from different subcellular organelles, such as endosomes or the Golgi apparatus. This Review discusses recent findings that GPCRs can signal from various subcellular locations, affecting different cellular responses. This research advances our understanding of GPCR signaling by showing that it is more complex and location specific than previously thought. In the future, these insights could lead to new treatments targeting specific GPCR pathways involved in diseases.

This summary was initially drafted using artificial intelligence, then revised and fact-checked by the author.

## Introduction

G-protein-coupled receptors (GPCRs) are among the largest families of transmembrane (TM) receptors, whose wide-ranging physiological functions include controlling the sympathetic nervous system as well as the perception of light, odor, taste and pain^1^. GPCR signaling is a fundamental process by which cells communicate with their extracellular environment and respond to signals such as hormones, neurotransmitters and other molecules. Upon ligand binding at the plasma membrane, GPCRs undergo a conformational change that initiates the activation of heterotrimeric G proteins, composed of Gα, Gβ and Gγ subunits, with the GPCR acting as a guanine nucleotide exchange factor for Gα. Depending on the identity of the Gα subunit, G proteins can control downstream signaling via intracellular second messengers including cyclic adenosine monophosphate (cAMP) and calcium ion (Ca^2+^), which then activate effector pathways to produce cellular responses. After G protein activation, GPCRs are phosphorylated by GPCR kinases (GRKs), followed by arrestin recruitment, which initiates GPCR internalization and the termination of signaling via clathrin-mediated endocytosis but can also trigger additional downstream signaling events such as through the mitogen-activated protein kinase (MAPK) pathway^2^.

In addition to the established role of GPCRs at the plasma membrane (that is, canonical GPCR signaling), growing evidence suggests that GPCRs mediate distinct signaling events at different subcellular locations beyond the plasma membrane, including the endosome, Golgi apparatus, endoplasmic reticulum and nucleus^3^. This spatially compartmentalized GPCR signaling is regulated by the localization of GPCRs to different endomembrane structures, either through subcellular trafficking or through lateral diffusion of GPCRs across different membrane locations^4^. The formation of GPCR signaling complexes can be differentially regulated by the lipid composition of these different endomembrane compartments, which produce specific molecular environments with unique allocations of various effector molecules, resulting in distinct regulation of GPCR signaling^5–7^. The context dependence of GPCR signaling contributes to functional diversity by tuning the dynamics and specificity of downstream signaling^8,9^. This emerging, noncanonical model of GPCR signaling has been extensively informed by the use of genetically encoded molecular tools such as fluorescent protein (FP)-based biosensors and pathway modulators, which allow real-time visualization and manipulation, respectively, of signaling pathway activities in living cells. In particular, the dedicated ability to selectively localize these tools to specific subcellular regions using various targeting sequences, combined with the inherent spatial precision of fluorescence microscopy, has proven ideally suited for investigating the architecture and function of spatially compartmentalized GPCR signaling^10^. In this Review, we first introduce various genetically encoded fluorescent biosensors and perturbation tools used to probe subcellular GPCR signaling. We then highlight several studies that utilized these tools to investigate the mechanism and function of spatially organized GPCR signaling in living cells.

## Genetically encoded fluorescent biosensors for direct visualization of subcellular location-specific GPCR signaling activity

Genetically encoded fluorescent biosensors have a common modular design composed of a sensing unit and reporting unit. The sensing unit determines the specificity of the biosensor for a target biochemical activity, such as by directly binding to a small molecule or being posttranslationally modified by an enzyme. Engagement of the sensing unit generally induces a change in biosensor conformation or localization, thereby altering the signal from the reporting unit, which converts the sensing unit-induced change in biosensor state into an optical readout and typically consists of one or more FPs. The exact readout depends on the configuration of the sensing and reporting units. For example, translocation-based biosensors containing a sensing unit fused to a single FP can be simply monitored by visualizing changes in the subcellular distribution of fluorescence. In other designs, the sensing unit is inserted into a single FP or between two FPs that serve as donor and acceptor for Förster resonance energy transfer (FRET), such that changes in the sensing unit conformation modulate FP spectral properties such as fluorescence intensity or FRET efficiency.

The ability to capture local GPCR signaling dynamics depends on the biosensor design strategy. Several biosensors incorporate intact, functional proteins or bind target molecules whose native localization behavior is inherited by the biosensor, thus providing spatial information. Alternatively, biosensors can be directly targeted to specific subcellular locations of interest by tagging with localization signals, including full-length proteins or short motifs that act as ‘molecular zip codes’. Below, we discuss various biosensor designs used to monitor GPCR activation and ligand binding dynamics, G protein coupling and signaling downstream of GPCRs, including second messengers and kinases.

### GPCRs and G proteins

GPCR signaling is initiated by the recognition of a specific ligand by its cognate receptor, followed by G-protein heterotrimer coupling^11^. Biosensors have been developed to monitor each step of this process based on conformational changes in intact proteins or protein–protein interactions between proteins of interest. In this section, we specifically discuss the design principles regarding FP-based biosensors for detecting ligand recognition, conformational dynamics and G protein coupling behavior of GPCRs, as well as their application in living cells. Other sensor classes, such as widely used bioluminescence energy transfer (BRET)-based biosensors (reviewed extensively by Pfleger et al. ^12^) are not described here.

#### Biosensors based on ligand-induced changes in GPCR conformation

Ligand binding to the extracellular region of a GPCR induces the rotation and outward displacement of TM6, accompanied by TM5 and TM7, which promotes G-protein heterotrimer coupling to transduce the extracellular signal^1^.

**Fig. 1:**
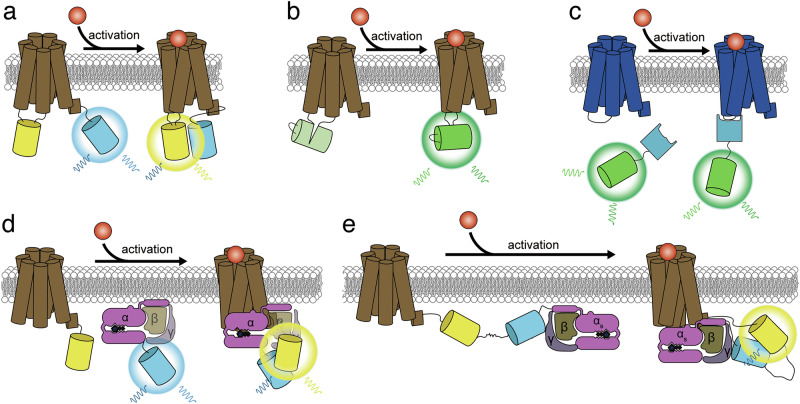
Fluorescent biosensors for detecting GPCR-G protein signaling. **a**,**b**, Biosensor designs based on ligand-induced conformational changes in GPCRs: donor and acceptor FPs forming a FRET pair are fused to the C-terminal tail and the third intracellular loop of a GPCR, respectively (**a**); a circularly permutated FP is inserted between the intracellular ends of TM5 and TM6 (**b**). **c**, A conformation-selective nanobody-based biosensor specifically translocates and binds to the active state of the GPCR. **d**,**e**, FRET-based biosensor designs relying on ligand-induced interactions between GPCRs and G proteins: GPCRs and G proteins are separately fused with donor and acceptor FPs (**d**); GPCRs and G proteins are linked via an ER/K linker, with donor and acceptor FPs positioned on either side of the linker (**e**).

In light of this activity-dependent conformational change, several biosensors have been designed that incorporate full-length GPCRs in the sensing unit, leveraging the movement of TM6 as a sensing unit molecular switch to modulate the fluorescence of a reporting unit. For example, FRET-based GPCR conformation biosensors were generated by inserting a donor FP (for example, CFP) into the third intracellular loop of a GPCR, which is the intracellular region most affected by TM 6 movement, and fusing an acceptor FP (for example, YFP) on the GPCR C-terminus, such that TM6 movement alters the proximity and orientation, and thus FRET, between the FPs (Fig. 1a). These sensors were successfully used to probe GPCR conformational dynamics in living cells and determine the pharmacological characteristics of target GPCRs including α2A-adrenergic receptor (α2AAR) and parathyroid hormone receptor^13^. Moreover, single-FP-based biosensors have been engineered by fusing the N- and C-termini of a circularly permuted FP (cpFP) to the intracellular ends of TM5 and TM6. This cpFP-based design has been used to develop sensitive indicators for monitoring the concentration, distribution and dynamics of GPCR ligands, particularly neurotransmitters and neuromodulators, including dopamine receptor (dLight and GRAB_DA_)^14^, α2-adrenergic receptor (GRAB_NE_)^15^ and muscarinic receptor (GRAB_ACh_)^16^ (Fig. 1b). cpFP-based biosensors can be engineered to exhibit very large fluorescence changes, allowing direct visualization of signaling dynamics in mouse brains. Importantly, as both FRET- and single-FP-based designs incorporate full-length GPCRs as the sensing unit, these biosensors will inherently adopt the native localization of the parent GPCR. Spatial information on GPCR activation can be obtained on the basis of the intrinsic localization of fluorescence intensity observed via fluorescence microscopy.

Another biosensor design for monitoring GPCR activation has also been developed that utilizes monomeric, single-domain antibodies produced in Camelid species, also called nanobodies. Because of their small size (~15 kDa) compared with conventional antibodies (~150 kDa), nanobodies can recognize previously inaccessible epitopes, including ones associated with specific protein conformations, and several nanobodies have been developed that selectively bind to and stabilize specific GPCR conformational states^17^. This unique ability was originally applied to study GPCR structural biology, which has proven extremely challenging due to the inherent conformational instability of GPCRs. As the inclusion of a nanobody helps to selectively purify the GPCR in a synchronized conformation, nanobodies greatly contributed to GPCR structural studies by helping to determine the structures of specific, activity-dependent conformations. The ability of nanobodies to bind to specific of GPCR conformations was subsequently used to develop conformational biosensors for the β2-adrenergic receptor (Nb80-green fluorescent protein (GFP))^18^ and opioid receptor (OR sensor)^19^ by tagging a nanobody that recognizes the desired conformation with an FP (Fig. 1c). These sensors allow direct visualization of the compartmentalized activation dynamics of GPCRs based on the subcellular localization of the fluorescence signal.

#### Biosensors based on GPCR and G protein coupling

G-protein heterotrimers engage in a multistep interaction cycle with GPCRs as a function of the guanine nucleotide-binding state of the Gα subunit. Activated GPCRs recruit G-protein heterotrimers and catalyze the conversion of GDP-bound Gα to the GTP-bound form. GTP-bound Gα then dissociates from the G-protein heterotrimer, Gα and Gβγ each subsequently interacting with their effectors to promote downstream signaling. The intrinsic guanosine triphosphatase (GTPase) activity of Gα results in the hydrolysis of bound GTP to GDP leading to reassembly of the heterotrimer and completing the GPCR interaction cycle^20^.

Multiple biosensor designs have been developed that leverage this dynamic G protein–GPCR interaction cycle to detect G protein coupling and activation. Early FRET-based versions were developed using a bimolecular design in which individual target proteins are tagged with either cyan fluorescent protein (CFP) or yellow fluorescent protein (YFP), and FRET between different protein pairs is monitored to reveal their interactions. This approach was applied to study the interactions among G protein subunits (for example, Gα, Gβ and Gγ), as well as between G proteins and various GPCRs (α2AAR, muscarinic acetylcholine receptor M4, A1 adenosine receptor and D2S dopamine receptor^21–24^) (Fig. 1d). However, the biosensor readout is highly dependent on co-localization of the tagged components, which was, in turn, sensitive to their relative expression. Furthermore, individual overexpression of FP-tagged G proteins or GPCRs may alter the endogenous coupling of the GPCR to its cognate Gα subunit by perturbing the balance of expression levels. Moreover, Malik et al. developed a series of biosensors based on systematic protein affinity strength modulation (SPASM) to detect G protein recruitment to activated receptors upon ligand stimulation^25^. These sensors are composed of a full-length GPCR and Gα subunit connected by a rigid ER/K helical linker that is sandwiched between a FRET FP pair (for example, CFP and YFP) and were used to probe coupling between multiple GPCRs (β2-, α1- and α2- adrenergic receptors, and adenosine type 1 receptor) and Gα subtypes (Gα_s_, Gα_i_ or Gα_q_). In contrast to FRET sensors that rely on the co-expression of FP-fused GPCRs and G proteins, SPASM sensors maintain 1:1 stoichiometry between the exogenously expressed GPCR and G protein. Moreover, because the ER/K linker forms an elongated structure consistent with a stable, extended persistence-length α-helix, this design provides significant spatial separation between the GPCR and G protein and clear contrast between the ‘on’ and ‘off’ conformations versus other G-protein-coupling sensors that utilize shorter, unstructured linkers (Fig. 1e).

Like the conformation-selective biosensors described above for probing the active GPCRs, the activation status of Gα proteins can be similarly captured by nanobodies. Specifically, Nb37 has been used to selectively visualize the active conformation of the stimulatory Gα protein (Gα_s_)^18^_._ Gα_s_ contains an alpha helical domain (AHD) and Ras-like GTPase domain (RHD); upon GPCR coupling, the AHD undergoes a major displacement relative to the RHD, which is a unique hallmark of Gα_s_ coupling to active GPCRs. Nb37 selectively binds this AHD-open conformation of Gα_s_, which is controlled by receptor-mediated GDP exchange. Live-cell fluorescence imaging of the subcellular redistribution of FP-tagged Nb37 has been successfully used not only to visualize acute Gα_s_ activation on the plasma membrane but also to reveal delayed Gα_s_ activation on endosomes.

### Downstream effectors of GPCR signaling

Numerous second messengers, kinases and other effectors are engaged to mediate cellular processes downstream of GPCR activation. These include Gα_s_- and Gα_i_-mediated cAMP production by adenylate cyclases (ACs), Gα_q_-mediated phosphoinositide regulation by phospholipase C, and Gα_12,13_-mediated RhoA regulation by guanine nucleotide exchange factors, which subsequently induce effects including cytosolic Ca^2+^ elevations and activation of protein kinase A (PKA), protein kinase C (PKC) and extracellular signal-regulated kinase (ERK)^1,2^. Many FP-based biosensors have been generated to monitor the dynamics of these downstream signaling components. In this section, we highlight the most commonly used biosensors for detecting GPCR-mediated cAMP–PKA and ERK signaling. Detailed discussion of biosensors for probing other GPCR-activated signaling cascades, including the Ca^2+^-mediated PKC and Gα_12,13_-mediated RhoA pathways, can be found in a recent review by Kim et al.^26^.

Importantly, these biosensors can be selectively targeted to discrete subcellular locations using various localization sequences fused to the N- or C-terminus. This includes the addition of short peptide sequences such as lipidation motifs from Lyn kinase or KRAS (plasma membrane), a nuclear localization signal, the phosphatidylinositol 3-phosphate-binding FYVE motif (early endosome) or the N-terminal region of DAKAP1 (mitochondrial surface) or cytochrome P450 (endoplasmic reticulum (ER) surface), as well as fusion to full-length proteins such as eNOS or giantin (Golgi surface), LAMP1 (lysosome surface or interior) and histone proteins (nucleus)^27^. Subcellular targeting greatly expands the scope of spatial signaling information that can be captured compared with GPCR- or G protein-based sensors, which adopt the intrinsic localization pattern of their sensing component. Furthermore, this strategy can be broadly applied to the various genetically encoded molecular tools introduced in this Review, including both biosensors and pathway modulators.

#### Biosensors detecting second messengers

GPCR activation triggers the production of second messengers that directly bind and activate effector proteins, thus amplifying signals. Effector proteins recognize the corresponding second messenger using highly selective binding pockets and usually undergo conformational changes upon binding. A common strategy for designing second-messenger biosensors therefore involves adoption of full-length or truncated effector proteins as the sensing unit to modulate the biosensor fluorescence readout^28^.

**Fig. 2:**
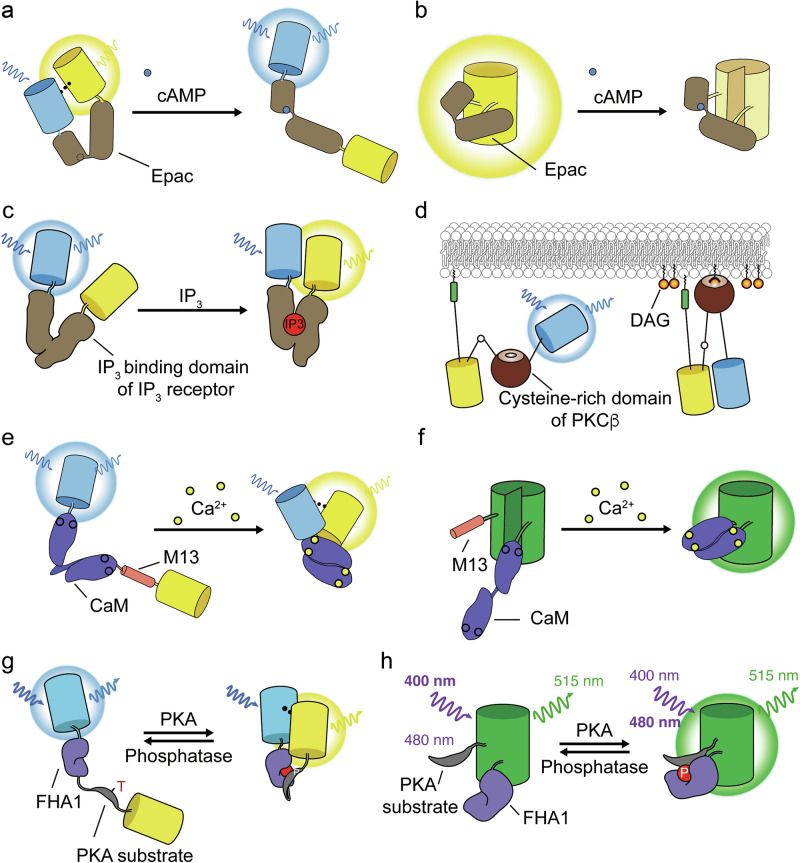
Biosensor designs for effectors of GPCR signaling. **a**, **b**, cAMP biosensors utilizing either FRET-based (**a**) or single FP-based (**b**) designs. sensors detect conformational changes in Epac upon cAMP binding. **c**, A FRET-based IP_3_ biosensor designed to monitor conformational changes in the IP_3_ binding domain of the IP_3_ receptor based on its IP_3_ binding status. **d**, A FRET-based DAG biosensor leveraging conformational changes in the cysteine-rich domain of PKC. **e**,**f**, Ca^2+^ biosensors utilizing either FRET-based (**e**) or single FP-based (**f**) designs. These sensors measure interactions between CaM and M13, dependent on the Ca^2+^-binding status of CaM. **g**,**h**, PKA kinase activity reporters utilizing either FRET-based (**g**) or single FP-based (**h**) designs. The fluorescence of these biosensors is dependent on the interaction between the PKA substrate and FHA1, based on the phosphorylation status of the PKA substrate domain.

cAMP is arguably the most well-known second messenger produced in response to GPCR activation. Intracellular levels of cAMP are carefully regulated by the local actions of ACs and phosphodiesterases, which respectively synthesize and degrade cAMP, as well as buffering by PKA biomolecular condensates, contributing to its spatial compartmentation^29^. cAMP signaling is mediated by various effector proteins, and designs of cAMP sensors thus adopt components from these effectors as their sensing units. The first genetically encoded cAMP sensor was derived from PKA and used the cAMP-induced dissociation of FP-tagged PKA catalytic (C) and regulatory (R) subunits to provide a FRET-based readout of cAMP dynamics^30,31^. Several unimolecular cAMP sensors have also been generated on the basis of PKA, utilizing either C- and R-subunit dissociation^32^ or cAMP-dependent conformational changes within the R subunit^33,34^ to modulate FRET or the fluorescence of a single FP. Alternatively, many if not most cAMP sensors are based on exchange protein directly activated by cAMP (Epac), a cAMP-dependent guanine nucleotide exchange factor that undergoes a hinge-like conformational change upon binding of cAMP. Epac isoforms have been routinely used over the years to generate FRET-based cAMP biosensors by sandwiching Epac between a pair of FPs, whereby the conformational change alters FP proximity and orientation (Fig. 2a^33,35–40^). The cAMP-mediated conformational change of Epac has also been used in several single-FP designs, with the conformational change altering the chromophore environment, resulting in a change in fluorescence intensity as a biosensor readout (Fig. 2b^41–44^). Other cAMP effectors, notably a bacterial cAMP-gated ion channel, have also been used to develop both FRET-based^45^ and single-FP^39^ cAMP sensors.

GPCR-mediated activation of phospholipase C triggers the hydrolysis of phosphatidylinositol 4,5-bisphosphate (PIP_2_) to produce inositol 1,4,5-triphosphate (IP_3_) and diacyl glycerol (DAG), which play central roles in modulating Ca^2+^ and PKC signaling. Diffusible IP_3_, which is released into the cytosol after PIP_2_ hydrolysis, induces ER Ca^2+^ release by directly binding IP_3_ receptors (IP_3_Rs) on the ER surface, while DAG remains embedded in the inner leaflet of the plasma membrane and can stimulate PKC membrane recruitment and activation. The IP_3_R comprises a family of Ca^2+^ release channels that govern the release of intracellular Ca^2+^ stores to initiate Ca^2+^ signaling events. IP_3_Rs specifically recognize IP_3_ through their IP_3_-binding domain, which has been utilized as a sensing unit in the development of IP_3_ biosensors. Typically, the binding domain is sandwiched between two FPs serving as donor and acceptor in a FRET-based reporting unit, such that IP_3_ binding will trigger a FRET change, enabling detection of physiologically relevant IP_3_ elevations in living cells^46,47^ (Fig. 2c). DAG-mediated PKC recruitment is regulated by the specific binding of DAG to C1 domains on conventional and novel PKC isoforms. DAG biosensors have been developed both by fusing a single FP to the C-terminus of a PKC C1 domain and by sandwiching a C1 domain between a FRET FP pair. Single-FP-based DAG biosensor report DAG production through biosensor translocation, monitored by fluorescence microscopy^44,48,49^. FRET-based DAG sensors, including Digda^50^ and Daglas^51^, were targeted to cellular membranes by tagging with subcellular targeting motifs, as discussed above. Membrane-anchored DAG biosensors undergo a conformational change upon DAG binding to the sensing unit, inducing a proximity change in the reporting unit that increases the FRET ratio of the biosensor (Fig. 2d).

IP_3_-triggered ER Ca^2+^ release downstream of GPCR activation is often a critical component of intracellular Ca^2+^ signaling, especially in nonelectrically excitable cell types. Like cAMP, Ca^2+^ is a ubiquitous second messenger involved in regulating countless cellular processes, typically through binding of the effector protein calmodulin (CaM). Ca^2+^-bound CaM (Ca^2+^/CaM) undergoes a dramatic conformational change when it interacts with Ca^2+^/CaM-binding regions in target proteins. Most genetically encoded Ca^2+^ indicators therefore use CaM together with a Ca^2+^/CaM-binding peptide derived from myosin light-chain kinase (for example, M13) as sensing unit receiver and switch domains. These two components are either tethered by a short, flexible linker and sandwiched between a pair of FPs to generated FRET-based sensors^52–55^ or inserted directly into an FP barrel in single-FP designs^56–63^. During intracellular Ca^2+^ elevations, Ca^2+^ binding to CaM induces intramolecular binding of the M13 peptide, triggering a conformational change in the biosensor that alters the fluorescence properties (for example, FRET efficiency and fluorescence intensity) of the reporting unit (Fig. 2e,f). Additional genetically encoded Ca^2+^ indicators have also been developed using an alternative design based on the Ca^2+^-binding protein troponin C as the sensing unit, yielding both FRET-based and single-FP sensors^64,65^.

#### Biosensors detecting effector activities

Among the major downstream effectors of GPCR signaling, protein kinases serve as key hubs to regulate diverse functional outputs. Kinases catalyze the phosphorylation of conserved substrate sequences in their target proteins; thus, genetically encoded kinase activity reporters (KARs) most commonly adopt a sensing unit design that incorporates a target-specific substrate peptide paired with a phosphoaminoacid binding domain (PAABD) as the receiver and switch domains, respectively^10^. KARs function as surrogate substrates for the kinase of interest, where the receiver domain substrate sequence gets phosphorylated by the active target kinase. This leads the PAABD switch domain to bind to the phopshorylated substrate, resulting in a conformational change in the sensing unit that alters the fluorescence output of the reporting unit.

PKA is one of the major effectors activated downstream of GPCR signaling pathways. cAMP binds to the PKA R subunits, unleashing the PKA C subunits to phosphorylate PKA substrate proteins and control various physiological processes, including cardiac and neuronal function. PKA localizes in the cytosol, but it also can be targeted to the cell membranes and other subcellular compartments through interactions with a family of scaffolding proteins known as A-kinase anchoring proteins (AKAPs), which assemble signaling complex together with GPCR and PKA regulators such as ACs and phosphodiesterases^29^. Thus, direct visualization of cAMP and PKA signaling dynamics is critical to fully understand the spatial organization of GPCR signaling events. The first genetically encoded A-kinase activity reporter (AKAR) was engineered by Zhang et al., who used a PKA consensus phosphorylation sequence with a 14-3-3τ PAABD as the sensing unit^66^. A second-generation sensor, AKAR2, swapped 14-3-3τ for an FHA1 domain, paired with a modified PKA substrate peptide^67^, which remains the sensing unit design found in almost all existing PKA sensors^68^.

In most cases, this bipartite PKA sensing unit is sandwiched by a pair of FPs such that the phosphorylation-induced interaction between the PKA substrate and FHA1 domains will result in a conformation change that modulates the biosensor FRET signal (Fig. 2g). However, other classes of PKA biosensor with distinct reporting strategies have also been developed. For example, Mehta et al. developed an excitation-ratiometric PKA biosensor (ExRai-AKAR) by conjugating the PKA substrate and FHA1 domain from FRET-based AKAR to the N- and C-termini of cpGFP^69^. This sensor displays two excitation peaks—at ~400 nm and ~500 nm—both of which result in emission at ~515 nm. A PKA activity-dependent conformational change, caused by binding of the FHA1 domain to the phosphorylated substrate, shifts the amplitudes of these two excitation peaks, with the ratio of fluorescence at each excitation wavelength providing a highly sensitive readout of PKA activity (Fig. 2h), with a much higher dynamic range versus the best-performing FRET-based PKA sensors. Further engineering of the sensor via linker optimization yielded an enhanced sensor, ExRai-AKAR2, with ~8-fold higher dynamic range than ExRai-AKAR, allowing the detection of PKA activity in the primary visual cortex of awake mice^70^.

The MAPK ERK, a master regulator of cell growth, proliferation and survival, is another critical downstream target of GPCR signaling. ERK activation requires the formation of a plasma membrane complex containing upstream MAPK components, including RAS, RAF and MEK, in response to growth factor receptor and GPCR signaling. Upon activation, ERK translocates from the membrane to the cytosol and nucleus, where it catalyzes the phosphorylation of various substrates and also regulates gene expression. A series of FRET-based ERK kinase activity reporters, or EKARs^71–74^, have been engineered over the years on the basis of the design heritage of AKAR. The sensing unit of FRET-based EKARs is composed of a peptide sequence, derived from the ERK substrate Cdc25C and containing a consensus MAPK target sequence (PRTP), and ERK docking sequence as the receiver domain, along with the proline-directed WW PAABD as the switch domain, which is sandwiched by two FPs as the reporting unit. Like AKAR, phosphorylation of the sensing unit by active ERK induces a conformational change that alters the proximity and orientation of the FPs to modulate FRET.

## Genetically encoded tools for location-selective perturbation of GPCR signaling

While genetically encoded biosensors allow the visualization of specific biochemical activities to precisely monitor spatially compartmentalized signaling, directly investigating the functional consequences of these local signaling activities is essential to achieve a comprehensive understanding of GPCR signaling. A variety of genetically encoded molecular tools are therefore being developed to directly and selectively perturb target signaling events as a necessary complement to the expanding biosensor toolkit. As with biosensors, these genetically encoded perturbation tools can be targeted to discrete subcellular locations using the aforementioned localization tags to allow location-selective modulation of signaling activities. Below, we discuss genetically encoded molecular tools such as peptide inhibitors and nanobodies that are being used to elucidate the spatial regulation of GPCR signaling.

### Peptide inhibitors

**Fig. 3:**
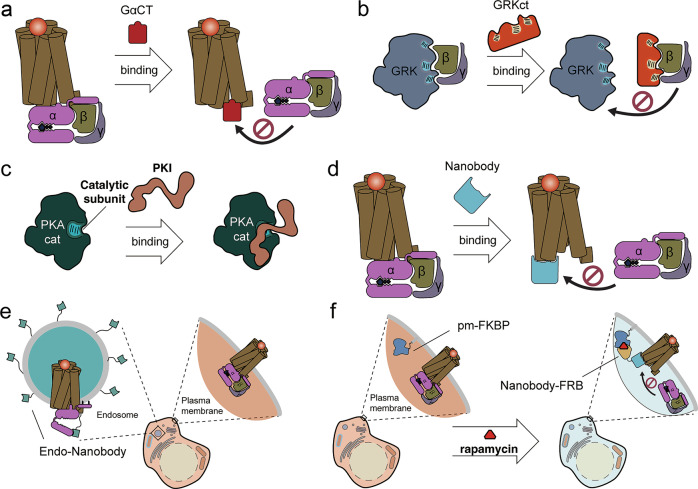
Genetically encoded tools for location-selective perturbation of GPCR signaling. **a**, The peptide inhibitor GαCT disrupts GPCR signaling by binding to the Gα protein interaction surface on the GPCR, thereby preventing GPCR–Gα coupling. **b**, The peptide inhibitor GRKct interferes with Gβγ signaling by blocking the interaction between Gβγ and GRKs. **c**, The peptide inhibitor PKI binds to the catalytic subunit of PKA (PKAcat), effectively inhibiting PKA activity. **d**, A conformation-specific nanobody targeting the active state of the GPCR perturbs G protein recruitment. **e**, Endosome-selective perturbation of GPCR signaling is achieved by fusing the conformation-specific nanobody with an endosome-targeting motif, restricting its action to endosomal compartments. **f**, Inducible plasma-membrane-selective perturbation of GPCR signaling involves targeting FKBP to the plasma membrane and fusing FRB to the C-terminus of a GPCR conformation-specific nanobody. Upon rapamycin treatment, FKBP and FRB heterodimerize, leading to the translocation of the nanobody to the plasma membrane for location-specific perturbation of GPCR signaling.

Peptide inhibitors are used to target critical regions involved in mediating interactions between signaling proteins. The overexpressed peptide docks with the target protein, occupying the binding surface and outcompeting interactions with endogenous effector, thus disrupting downstream signal transduction. These peptides are often designed on the basis of structural information to mimic the binding surface of an endogenous binding partner, while lacking functional effector domains to avoid unwanted activity. For example, the C-terminus of Gα is a critical region involved in coupling to GPCRs. Thus, Gα peptide inhibitors were developed by screening a library of peptide fragments spanning various regions of the C-terminal tail from different Gα isoforms. As a consequence, 55- and 83-amino acid fragments derived from the C-terminal tails of Gα_q_(ref. ^75^) and Gα_s_(ref. ^76^), respectively, were engineered as peptide inhibitors for their respective Gα isoforms (Fig. 3a). Similarly, the free Gβγ dimer can directly recruit GRK to phosphorylate GPCRs and initiate receptor desensitization. This interaction also directly inhibits Gβγ signaling independent of GRK kinase activity^77^. Koch et al. identified the critical region within the GRK–Gβγ binding interface by screening GRK truncation mutants of various lengths. Consequently, they showed that GRKct, a single, 28-residue peptide derived from the GRK C-terminus, effectively inhibited Gβγ-mediated GRK activation^78^ (Fig. 3b). Peptide inhibitors can also be used to disrupt enzyme–substrate interactions. For example, the PKA inhibitor (PKI) is a heat-stable inhibitory protein, originally identified in skeletal muscle^79^, that blocks PKA activity using its N-terminal pseudosubstrate domain, which mimics the structure of the type I PKA regulatory subunit^80^. Based on detailed interaction models showing that an N-terminal PKI fragment (5–24) forms a sandwich-like structure with PKA catalytic subunit residues Tyr235 and Phe239, this active domain of endogenous PKI (5–24) was engineered to be utilized as a potent synthetic peptide inhibitor (Fig. 3c).

### Nanobodies

The diverse signaling states mediated by multiple different ligands acting through a single GPCR stems from the various conformations each receptor adopts in response to ligand binding. The degree of conformational change within individual GPCRs controls a wide range of functional outputs, including activation or inhibition of downstream signaling pathways. As mentioned in the ‘GPCRs and G proteins’ section, nanobodies are capable of recognizing and stabilizing specific conformations of their target proteins. In addition to their application as molecular tools to detect active GPCR conformations in cells, nanobodies can alternatively be utilized to trap target GPCRs in specific conformations and perturb GPCR signaling when expressed at high levels in cells, similar to how ligands stabilize specific conformations to control GPCR activity. For example, Staus et al. investigated the ability of 18 different nanobodies, which were originally generated to capture β_2_AR conformations for structural studies, to regulate downstream signaling events. They observed a varying range of inhibitory effects on cAMP production, GRK-mediated receptor phosphorylation, arrestin recruitment and endocytosis through either steric blockade of downstream effector interactions or stabilization of inactive receptor conformations^81^. Similarly, Nb80, which recognizes active βARs, can be overexpressed to competitively block Gα_s_ coupling, because Nb80 specifically recognizes and binds a cytosolic interface present in the ligand-bound conformation of βARs, which is a critical region for Gα_s_ coupling (Fig. 3d). Taking advantage of a well-characterized system for chemically induced dimerization, Irannejad et al. used FKBP and FRB fused to plasma membrane or Golgi targeting sequences and Nb80, respectively^82^, to dynamically recruit Nb80 to either compartment and show that selectively blocking Gα_s_ coupling at the Golgi strongly reduced adrenergic activation of cAMP production (Fig. 3f). We also fused two tandem copies of the endosome-targeting FYVE domain to the C-terminus of Nb37, which stabilizes Gα_s_ in the open conformation, to characterize the effect of chronic location-specific perturbation on downstream signaling events^83^ (Fig. 3e).

## Spatiotemporal regulation of GPCR signaling

GPCRs transduce extracellular signals to multiple different subcellular locations. Although the major site of receptor localization is the plasma membrane, where extracellular ligands are recognized at the cell surface, emerging evidence suggests that GPCRs are present at multiple subcellular locations, although the signaling capacity and physiological role of these subcellular receptor pools are not clearly understood. Simultaneous visualization and perturbation of localized signaling through combined application of subcellularly targeted biosensors and perturbation tools is critical for the comprehensive understanding of GPCR signaling.

### Improved biosensors distinguish GPCR signaling within plasma membrane microdomains

GPCRs are expressed primarily on the plasma membrane, which is spatially segmented into microdomains characterized by their distinct lipid compositions. Nanometer-scale, sphingolipid- and cholesterol-enriched membrane microdomains called lipid rafts are dynamically regulated and frequently implicated in cell signaling, often promoting the formation of signaling complexes. Given that GPCR signaling components have been shown to localize differentially between lipid raft and nonraft regions, the ability to investigate GPCR signaling activities within specific microdomains can provide essential insights into the role of lipid rafts in regulating GPCR signaling.

**Fig. 4:**
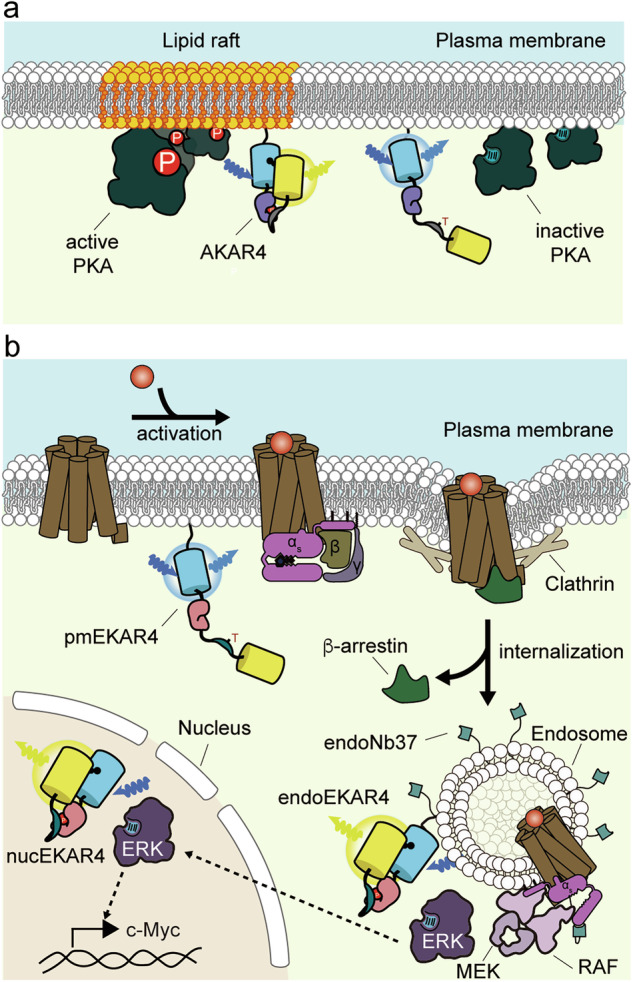
Schematic illustration of the biological applications of biosensors in investigating the spatial organization of cell signaling. **a** Plasma membrane microdomain-specific PKA activity is assessed using FRET-based PKA activity reporters targeted to lipid raft and nonlipid raft regions. The results demonstrate higher basal PKA activity within lipid rafts compared with nonlipid raft microdomains. **b** The spatial organization of GPCR-mediated ERK activity is analyzed using ERK kinase activity reporters targeted to the plasma membrane, endosomes and nucleus. The findings reveal that GPCR-mediated ERK activity originates from endosomes, not the plasma membrane, and subsequently propagates to the nucleus.

Dynamic range is a critical factor in determining whether a biosensor is able to detect subtle changes in the target biochemical activity. Combined with precise targeting strategies, efforts to improve biosensor dynamic range have been essential for sensitively exploring distinct biochemical activities at the suborganelle level, such as within plasma membrane microdomains. For example, by developing a fourth-generation FRET-based A-kinase activity reporter (AKAR4), with almost double the dynamic range of the prior version, Depry et al. were able to identify distinct GPCR-mediated PKA activity from lipid raft and nonraft plasma membrane regions. The sensor was targeted to lipid rafts via fusion to an N-terminal motif from Lyn kinase, which undergoes myristoylation and palmitoylation to enable anchoring to the inner leaflet of the plasma membrane, as well as to nonraft regions via C-terminal fusion of the polybasic sequence and CAAX motif derived from KRAS, which is a critical region governing prenylation and membrane localization of KRAS. This strategy revealed the presence of higher basal PKA activity in lipid raft microdomains compared with nonraft regions, which depended on raft integrity as well as PKA localization. Moreover, they found a role for lipid rafts in controlling GPCR-mediated PKA activity by identifying the negative effect of raft microdomains on adrenergic-stimulated plasma membrane PKA activity^84^ (Fig. 4a). The raft-targeted sensor also exhibited slower phosphatase mediated dephosphorylation upon PKA inhibition, hinting at distinct temporal dynamics of raft and nonraft PKA activity. This study highlights the spatial resolution of FRET-based biosensors to distinguish target activities at not only the subcellular but also the submicrometer level. Moreover, this work inspired follow-up studies utilizing different approaches that further contributed to our understanding of how membrane microdomains regulate receptor dimerization or the recruitment of various effectors, including arrestin.

### Biosensors illuminate endosome-localized GPCR signaling regulated by receptor trafficking

GPCR signaling occurs not only at the plasma membrane but also from the endosome membrane following receptor endocytosis. Several GPCRs, including thyroid-stimulating hormone receptor (TSHR), show different downstream signaling outputs depending on whether the receptor-mediated signaling originates from the plasma membrane or endosomes. To study GPCR-endocytosis-dependent cAMP production at endosomes, Calebiro et al. generated engineered mice ubiquitously expressing a FRET-based cAMP reporter so that they could monitor real-time cAMP production in thyroid follicles derived from the transgenic mice^85^. Biosensor imaging revealed that the endocytosed receptor is capable of transducing signals from the endosome upon thyroid-stimulating hormone stimulation. Specifically, by monitoring the kinetics of the biosensor response with and without application of an endocytosis inhibitor, they were able to reveal persistent cAMP production originating from endosome-localized TSHR, in contrast to transient signaling from plasma membrane TSHR.

Similarly, biosensor imaging identified that β_2_AR also initiates signaling at the endosome following ligand-stimulated endocytosis, resulting in a different signaling output versus plasma-membrane-originated signaling. To study the spatiotemporal dynamics of β_2_AR activity, Irannejad et al. performed real-time visualization of the translocation of diffuse, GFP-conjugated Nb80 to subcellular locations in cells expressing β_2_AR by examining the colocalization of fluorescence signals from Nb80 and labeled β_2_AR^18^. Upon adrenergic stimulation, acute translocation of diffuse GFP-conjugated Nb80 to the plasma membrane was observed, leading to colocalization of Nb80 and β_2_AR. GFP-conjugated Nb80 did not colocalize with β_2_AR-containing clathrin-coated pits that formed a few minutes after stimulation, suggesting that arrestin-mediated endocytosis terminates plasma-membrane-localized β_2_AR activation. However, Nb80 was observed to relocalize to β_2_AR-containing endosomes after clathrin-mediated endocytosis, suggesting the existence of an active pool of β_2_AR on endosomes that signals independently of the plasma membrane. By perturbing endosomal signaling using an endocytosis inhibitor, the authors identified a specific role for cAMP production mediated by this endosomal pool of active β_2_AR in controlling local CREB phosphorylation and PKAcat nuclear entry^5,6^. Moreover, phosphoproteomics revealed that endosome-originated β_2_AR signaling selectively controls the phosphorylation of a subset of PKA substrate proteins distinct from those regulated by PM-originated signaling^86^. These studies demonstrate the utility of biosensors for investigating endosomal GPCR signaling and revealed the role of receptor endocytosis in sorting GPCR signals by controlling temporal dynamics to regulate specific signaling outputs.

### Genetically encoded tools highlight distinct intracellular GPCR pools

Some GPCRs constitutively localize not only to the plasma membrane but also to various intracellular membranes, including the Golgi apparatus or sarcoplasmic reticulum. Utilizing an Nb80-based adrenergic receptor active conformation indicator, Irannejad et al. discovered the existence of active β_1_AR on the Golgi membrane upon stimulation with a β_1_AR-specific ligand through Nb80 colocalization with a Golgi marker. Combining endocytosis inhibitor treatment with biosensor imaging, they were able to determine that the Golgi and plasma membrane β_1_AR pools are functionally separate by showing that inhibition of receptor endocytosis had a negligible effect on Golgi receptor activity. They also revealed that plasma-membrane-originated PKA signaling showed faster activation kinetics compared with the Golgi. Moreover, they selectively inhibited Golgi-localized β_1_ARs via chemically inducible Golgi targeting of Nb80 and found that the Golgi pool of the receptors contributes to intracellular cAMP production and local PKA activation^82^. Nash et al. similarly used Nb80 translocation imaging to investigate local β_1_AR activity in cardiomyocytes. Adrenergic stimulation of the cAMP-mediated PIP4 hydrolysis pathway is one of the critical signaling pathways involved in the development of cardiac hypertrophy. By investigating Nb80 translocation and colocalization with subcellular location markers, combined with inhibitor treatments, Nash et al. found that Golgi-localized β_1_AR signaling is responsible for triggering cAMP-mediated PIP4 hydrolysis in cardiomyocytes through the formation of local PLCε and EPAC complexes^87^. Moreover, Wang et al. discovered that β_1_AR is also present on the sarcoplasmic reticulum membrane and can be activated via the monoamine transporter OCT3 by utilizing biochemical approaches and genetically modified mice lacking OCT3 expression. By imaging SR-targeted AKAR in response to adrenergic stimulation, they demonstrated that this SR-localized β_1_AR pool can locally activate PKA, which eventually controls myocyte contraction in mouse ventricular cardiomyocytes^88^. Given the distinct subcellular expression of β_1_AR at the plasma membrane and intracellular membranes including the Golgi and SR, as well as the fact that these pools do not interact with each other, perturbing the correct β_1_AR pool is critical to achieving meaningful clinical outcomes. The combination of biosensor imaging and molecular tool-mediated perturbation of local signaling allows investigations into the intracellular membrane-originated signaling that were previously infeasible using chemical inhibitors and suggests a critical therapeutic concept regarding the location bias of intracellular of GPCR pools to unlock desired effects.

### Genetically encoded tools reveal spatially organized GPCR signaling across different subcellular locations

As a downstream effector of GPCR signaling, ERK controls target gene expression in the nucleus. Yet, precisely how GPCRs control this local ERK activity has remained unclear, especially in light of recent findings that GPCRs can be activated not only at the plasma membrane but also at the endosome membrane after ligand-stimulated endocytosis. In the canonical model of GPCR-mediated ERK activation, arrestin plays a key role in regulating ERK activation by scaffolding different MAPK pathway components including RAS, RAF and MEK to form a signaling complex, while the role of GPCR endocytosis, which is a consequence of arrestin recruitment to GPCRs, in regulating this pathway has been controversial^89–91^. Given that clathrin-mediated GPCR endocytosis substantially alters the biochemical environment surrounding GPCRs in terms of lipid composition, as well as the local concentration of signaling proteins, including critical components for recruiting MAPK pathway regulators, combining direct measurement of GPCR-mediated ERK activation and selective perturbation of GPCR activity in different subcellular compartments can provide a more complete understanding of the spatial organization of this signaling pathway. To this end, we utilized subcellularly targeted EKAR to detect GPCR-mediated ERK activity at the plasma membrane, endosome and nucleus upon β_2_AR activation^83^. Notably, the overall kinetics of GPCR-mediated ERK activity were slower than those observed upon growth factor stimulation. Surprisingly, we observed no detectable increase in plasma-membrane-localized ERK activity upon adrenaline stimulation. By contrast, endosome-targeted EKAR revealed strong ERK activation, which was found to depend on receptor endocytosis, suggesting spatially compartmentalized GPCR-mediated ERK activation. However, because upstream MAPK components such as RAS, RAF and MEK are present on the plasma membrane, further investigations are needed to assess whether plasma-membrane-localized ERK activity is triggered by other types of GPCR.

The canonical role of arrestin has also been challenged by studies showing that GPCR signaling can still activate ERK in cells genetically modified to lack arrestin expression^89,90^. Alternatively, Gα_s_ may be a critical factor in regulating endosomal GPCR-mediated ERK activity, because Gα_s_ has been shown to reassociate with endosome-localized GPCRs, whereas arrestins only transiently interact with and quickly dissociate from class A GPCR-containing endosomes. To examine the role of Gα_s_ in endosomal GPCR-mediated ERK activity, we further combined EKAR imaging with genetically encoded endosome-targeted tools, including GsCT and Nb37, to selectively perturb endosome-localized Gα_s_ activity^83^. We found a direct correlation between endosome-selective perturbation of Gα_s_ and endosomal ERK activity, suggesting that endosome-localized Gα_s_ is the critical upstream regulator of endosome-localized ERK activity. By imaging nuclear-targeted EKAR in conjunction with endosome-targeted Nb37, GsCT and Nb80, we further revealed that this endosomal GPCR–Gα_s_ signaling axis directly leads to nuclear ERK signaling. Moreover, by selectively blocking this endosomal signaling axis using endosome-targeted GsCT, we revealed that this spatially organized GPCR signaling regulates ERK target gene expression and cell proliferation (Fig. 4b). This study illustrates the power of combining targeted biosensors and genetically targeted perturbations to simultaneously elucidate the role of different subcellular compartments in organizing spatially regulated signaling architectures and unravel their cellular functions.

## Conclusion and future perspectives

How spatially biased GPCR signaling enables selective regulation of certain downstream pathways over others has increasingly been the subject of extensive study. Subcellularly targeted molecular tools, including fluorescent biosensors and engineered perturbation tools, have been instrumental in these efforts by allowing direct visualization and characterization of spatiotemporally compartmentalized GPCR signaling. Further advances in our understanding of compartmentalized GPCR signaling networks will similarly require development of more advanced tools and methodologies. One emerging strategy monitors compartmentalized signaling by using spontaneous fragment complementation to target sensors to endogenously expressed proteins within subcellular microdomains^92^. Fluorescent sensors targeted to endogenous proteins (FluoSTEPs) are generated by inserting GFP_11_, a fragment of spontaneously complementing split GFP, into the endogenous gene locus of a target protein of interest via genome-editing and overexpressing the other fragment of split GFP, GFP_1–10_, fused to the biosensor as the FRET donor. An advantage of this approaches over current strategies, which involve fusing targeting motifs directly to molecular tools, is the opportunity to reduce nonspecific contamination of biosensor signals and minimize off-target effects caused by occasional mislocalization outside the desired region. Indeed, FluoSTEPs greatly reduce these artifacts because the functional (that is, fluorescent) biosensor is only produced in cells following reconstitution of the split GFP fragments at the specific protein of interest, resulting in more precise biosensor targeting. Moreover, targeting molecular tools to unique cellular structures such as clathrin-coated pits and biomolecular condensates can be achieved without disrupting the stoichiometry of these assemblies. However, the dynamic range of FluoSTEPs is relatively small compared with conventional FRET-based biosensors. Further optimization of the FluoSTEP design and improvement of the dynamic range, while retaining the capability for endogenous protein targeting, will allow more sensitive examination of native compartmentalized GPCR signaling. Moreover, subcellular targeting of molecular tools for perturbing specific subcellular signaling pathways could also inherit the strategy of FluoSTEPs.

Another exciting advance is the ability to multiplex multiple different target signaling events within a single cell. While the impact of GPCR location bias has been extensively studied with respect to individual downstream pathways, simultaneous imaging of different biosensors in a single cell would enable a richer understanding of how spatial compartmentation shapes GPCR signaling networks. Chemigenetic sensor designs that leverage fluorescent dyes and self-labeling protein tags, such as the HaloTag protein, show particular promise in this regard. The spectral flexibility of this approach enables biosensors to readily expand into the far-red spectral region, as illustrated by a chemigenetic PKA activity reporter, HaloAKAR, that enabled robust five-color multiplexed imaging of signaling activities in living cells^93^. Moreover, HaloTag variants exhibit distinct fluorescence lifetimes when labeled with different rhodamine dyes^94^. This property enabled fluorescence lifetime-based multiplexed imaging of three distinct targets within a single spectral channel, supporting up to six-species multiplexing across two spectral channels. Although this strategy has not yet been integrated with biosensors, future efforts to leverage the spectral versatility and lifetime diversity of HaloTag holds significant potential to greatly expand the capabilities of biosensor multiplexing, allowing more comprehensive and simultaneous investigation of complex biological systems.

Advances in our ability to precisely target biosensors and molecular perturbations to specific subcellular locations or proteins of interest, combined with more powerful and flexible techniques for multiplexed biosensor imaging to monitor multiple signaling events simultaneously, will pave the road toward a greater understanding of the spatial organization of GPCR signaling and its cellular functions. New insights gained through these approaches will ultimately help to establish spatially biased GPCR signaling as a critical target for developing new therapeutic approaches for GPCR-related diseases.
